# Characterization of the Efflux Capability and Substrate Specificity of Aspergillus fumigatus PDR5-like ABC Transporters Expressed in Saccharomyces cerevisiae

**DOI:** 10.1128/mBio.00338-20

**Published:** 2020-03-24

**Authors:** Brooke D. Esquivel, Jeffrey M. Rybak, Katherine S. Barker, Jarrod R. Fortwendel, P. David Rogers, Theodore C. White

**Affiliations:** a Division of Cell Biology and Biophysics, School of Biological Sciences, University of Missouri—Kansas City, Kansas City, Missouri, USA; b Department of Clinical Pharmacy and Translational Science, University of Tennessee Health Science Center, Memphis, Tennessee, USA; University of Melbourne

**Keywords:** *Aspergillus fumigatus*, efflux, drug resistance, filamentous fungi, ABC transporter, *PDR5*, efflux inhibitor

## Abstract

This research analyzed six Aspergillus fumigatus genes encoding putative efflux proteins for their roles as transporters. Th*e* A. fumigatus genes *abcA, abcC, abcF, abcG, abcH*, and *abcI* were cloned into plasmids and overexpressed in a Saccharomyces cerevisiae strain in which the highly active endogenous ABC transporter gene *PDR5* was deleted. The activity of each transporter was measured by efflux of rhodamine 6G and accumulation of alanine β-naphthylamide. The transporters AbcA, AbcC, and AbcF had the strongest efflux activities of these compounds. All of the strains with plasmid-expressed transporters had more efflux activity than did the *PDR5*-deleted background strain. We performed broth microdilution drug susceptibility testing and agar spot assays using an array of compounds and antifungal drugs to determine the transporter specificity and drug susceptibility of the strains. The transporters AbcC and AbcF showed the broadest range of substrate specificity, while AbcG and AbcH had the narrowest range of substrates. Strains expressing the AbcA, AbcC, AbcF, or AbcI transporter were more resistant to fluconazole than was the *PDR5*-deleted background strain. Strains expressing AbcC and AbcF were additionally more resistant to clotrimazole, itraconazole, ketoconazole, and posaconazole than was the background strain. Finally, we analyzed the expression levels of the genes by reverse transcription-quantitative PCR (RT-qPCR) in triazole-susceptible and -resistant A. fumigatus clinical isolates. All of these transporters are expressed at a measurable level, and transporter expression varied significantly between strains, demonstrating the high degree of phenotypic variation, plasticity, and divergence of which this species is capable.

Our objective was to determine if these undercharacterized proteins function as efflux transporters and then to better define whether their efflux substrates include antifungal drugs used to treat fungal infections. We chose six A. fumigatus potential plasma membrane ABC transporter genes for analysis and found that all six genes produced functional transporter proteins. We used two fungal systems to look for correlations between transporter function and drug resistance. These transporters have the potential to produce drug-resistant phenotypes in A. fumigatus. Continued characterization of these and other transporters may assist in the development of efflux inhibitor drugs.

## Abstract (precis)

One mechanism behind drug resistance is altered export out of the cell. This work is a multifaceted analysis of membrane efflux transporters in the human fungal pathogen A. fumigatus. Bioinformatics evidence infers that there is a relatively large number of genes in A. fumigatus that encode ABC efflux transporters. However, very few of these transporters have been directly characterized and analyzed for their potential role in drug resistance. Our objective was to determine if these undercharacterized proteins function as efflux transporters and then to better define whether their efflux substrates include antifungal drugs used to treat fungal infections. We chose six A. fumigatus potential plasma membrane ABC transporter genes for analysis and found that all six genes produced functional transporter proteins. We used two fungal systems to look for correlations between transporter function and drug resistance. These transporters have the potential to produce drug-resistant phenotypes in A. fumigatus. Continued characterization of these and other transporters may assist in the development of efflux inhibitor drugs.

## INTRODUCTION

The filamentous fungal pathogen Aspergillus fumigatus is the most common cause of invasive mold infection in humans and is associated with an alarmingly high mortality rate (1). Currently available antifungal drugs to treat invasive aspergillosis are very limited, either due to issues with safety and toxicity to the host or because they have narrow modes of action leading to the potential for the development of drug resistance (2–4). In addition, filamentous fungi are oftentimes intrinsically resistant to antifungals that are commonly used to treat other types of fungal infection, as is the case with A. fumigatus resistance to fluconazole (FLC) (5–7).

In many well-studied fungal pathogens, multidrug resistance is thought to be caused by the overexpression or increased activity of fungal plasma membrane transporters (8–11). Commonly, the transporters belong to the ATP binding cassette (ABC) superfamily of proteins and use ATP hydrolysis as a source of energy to export a broad range of substrates including, but not limited to, antifungal drugs across biological membranes (11–16). While the number of transporter genes within genomes is varied, and the gene sequences between species can be extremely diverse, there are several characteristic ABC transporter motifs that are conserved across organisms. The hallmark structures of ABC transporters include nucleotide-binding domains (NBD) that bind ATP and transmembrane domains (TMD) that are thought to play a role in substrate recognition and specificity (13, 17, 18). The number, arrangement, and topology of these domains can vary within and between organisms. The multiplicity of fungal ABC transporters allows for a diversity of physiological functions that go beyond membrane transport and drug resistance (13, 17, 19–22).

The best-characterized fungal ABC transporter is that encoded by the *PDR5* gene in the yeast Saccharomyces cerevisiae (23). The overexpression of *PDR5* leads to resistance to a number of structurally unrelated drugs, while the deletion of *PDR*5 leads to hypersusceptibility to these drugs (21). More recently, the overexpression of transporters in filamentous fungi, particularly Pdr5-like proteins, is being recognized as a major threat to clinical treatment success (reviewed in reference 24).

In A. fumigatus specifically, AbcA, AbcB, AtrF, and other ABC transporters have been found to be overexpressed in drug-resistant A. fumigatus clinical isolates (25, 26). AbcC (also known as Cdr1B and AbcG1) is one of the better-characterized A. fumigatus ABC transporters, and the deletion of the gene encoding this protein in A. fumigatus can reverse azole drug resistance (25). Gene transcripts for AbcA, AbcC, and other A. fumigatus ABC transporters have been shown to be upregulated upon azole drug treatment and may also play a role in virulence (27, 28).

Previous analysis revealed measurable, energy-dependent efflux of fluconazole in A. fumigatus and the plant pathogen Magnaporthe oryzae (29, 30). Filamentous fungi contain a higher number of genes encoding predicted ABC transporters than do yeast species such as Saccharomyces cerevisiae, Candida albicans, Cryptococcus neoformans, Candida glabrata, and Candida krusei (13, 14, 17, 18). However, very few of these genes have been directly demonstrated to encode functional transporters, and even fewer genes have been analyzed for their potential role in drug resistance (13, 17).

Clorgyline has recently been identified as a possible inhibitor of energy-dependent efflux in various fungi. It inhibits the ABC efflux pumps CaCdr1p and CaCdr2p that are responsible for significant azole resistance in C. albicans (31). It reverses FLC resistance in S. cerevisiae strains expressing C. albicans and C. glabrata ABC transporters (31). Clorgyline also inhibits energy-dependent efflux of [^3^H]FLC in *M. oryzae* (30). These results indicate that the mechanism of transport inhibition by clorgyline is broadly conserved throughout yeast and filamentous fungi.

The focus of this research is to directly characterize A. fumigatus genes that have been putatively identified to encode ABC efflux transport proteins. This includes analyses to determine transporter functionality, substrate specificity, and potential roles in drug resistance. There are multiple challenges to a study of this nature, including the presence of a relatively high number of encoded transporters in the A. fumigatus genome and the fact that A. fumigatus is not typically a model organism. The presence of multiple introns and the large transporter gene size (many are ∼4,500 bp) in the A. fumigatus genome add further complications to molecular-based experiments (32, 33). In addition, the filamentous and nonhomogeneous growth of molds, as well as the variety of morphotypes exhibited as the cells develop makes drug susceptibility testing hard to standardize and the analysis of growth characteristics difficult (34).

For a more straightforward approach to study the function of individual A. fumigatus genes, we created recombinant Saccharomyces cerevisiae strains with inducible overexpression of individual transporters from A. fumigatus. The controlled overexpression of A. fumigatus genes in an S. cerevisiae model allows the functional analysis of transport activity of Pdr5-like putative ABC transporters from an important human pathogen. The efflux-deficient S. cerevisiae host strain provided a more sensitive assay for pump activity of the A. fumigatus genes to be analyzed in a background strongly depleted of endogenous pump activity. It also allowed a direct comparison and contrast of the different A. fumigatus ABC transporters.

## RESULTS

### Inhibition of azole efflux by clorgyline.

**FIG 1:**
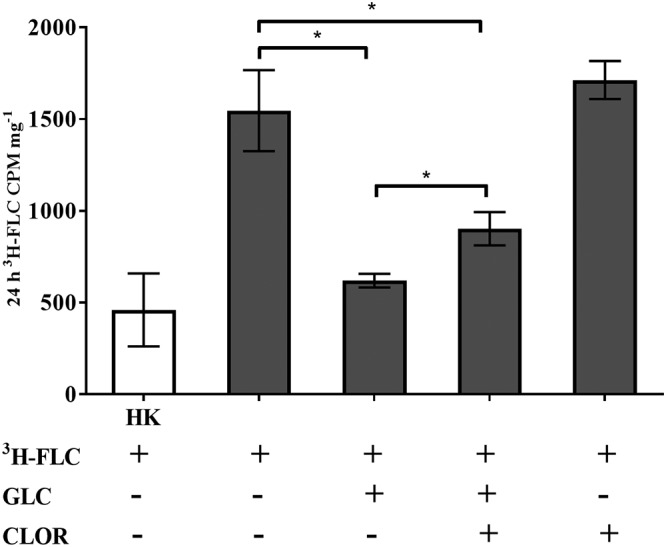
Energy-dependent azole efflux inhibition with clorgyline. Intracellular [^3^H]FLC concentration was measured in cells that were treated with [^3^H]FLC alone (19.25 nM), [^3^H]FLC with 2% glucose (efflux active), [^3^H]FLC with 2% glucose and clorgyline (233 μM), and with [^3^H]FLC with clorgyline. Heat killed (HK) represents the nonspecific, background-level [^3^H]FLC accumulation as the cells have been killed at 95°C for 10 min. [^3^H]FLC accumulation was measured after 20 h under all conditions. Error bars represent the standard deviation of biological triplicates for each condition.

To examine the effect of clorgyline as a possible inhibitor of energy-dependent efflux in A. fumigatus strain AF293, we treated energized, efflux-active cells with either [^3^H]FLC alone (+[^3^H]FLC +GLC) or [^3^H]FLC with clorgyline ([^3^H]FLC +GLC +CLOR). We compared the results to [^3^H]FLC treatment in deenergized, efflux-inactive cells with and without clorgyline (+[^3^H]FLC –GLC +CLOR and +[^3^H]FLC –GLC, respectively), as well as heat-inactivated background control (HK) cells (Fig. 1).

After 20 h of incubation, [^3^H]FLC uptake was observed in both deenergized and energized cells, with the energized cells showing significantly reduced intracellular [^3^H]FLC concentration compared to that of deenergized cells (Fig. 1, −GLC versus +GLC samples). The reduced azole accumulation in the energized cells is most likely the result of the activation of energy-dependent efflux pumps transporting the [^3^H]FLC out of the cell. However, when the energized, efflux-active cells were treated with clorgyline (+[^3^H]FLC +GLC +CLOR), there was a significant increase in intracellular [^3^H]FLC accumulation, indicating that energy-dependent efflux was at least partially prevented in the presence of clorgyline. Clorgyline had no effect on [^3^H]FLC accumulation in efflux-inactive cells (+[^3^H]FLC –GLC +CLOR). These results demonstrated a need to investigate A. fumigatus ABC transporters. As described below, the expression of each gene in S. cerevisiae allows the study of each gene individually for its role in drug resistance.

### Drug susceptibility.

Broth microdilution was used to determine the MIC_80_ for a variety of antifungal compounds in the S. cerevisiae strains expressing A. fumigatus transporters (Table 1). The BΔ*PDR5* strain carrying an empty plasmid was used as a control. In Table 1, changes in the drug susceptibilities of the strains expressing A. fumigatus genes compared to the control strain are highlighted in green (increased resistance) or blue (increased susceptibility).

**TABLE 1:**
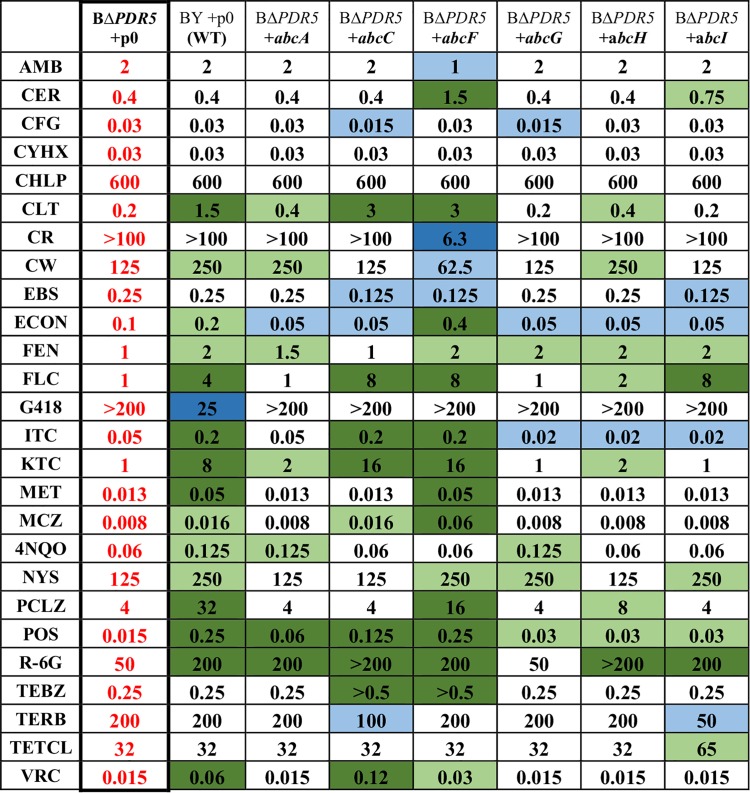
Broth microdilution drug susceptibility of strains expressing A. fumigatus transporters^*a*^

a Drug abbreviations are described in Materials and Methods. BΔ*PDR5* denotes the BY4741 strains with a *PDR5* gene deletion. BY +p0 denotes the wild-type background strains BY4741 with an empty pYES plasmid. +*abcX* denotes the A. fumigatus gene expressed from a pYES plasmid. Dark-green boxes highlight 4× or greater increased resistance, and light-green boxes highlight 2× increased resistance. Dark-blue boxes highlight 4× or greater increased susceptibility, and light-blue boxes highlight 2× increased susceptibility.

Strains expressing *abcC* and *abcF* both showed an increased MIC_80_ for many of the azoles tested, including clotrimazole (CLT), FLC, itraconazole (ITC), ketoconazole (KTC), posaconazole (POS), and tebuconazole (TEBZ), indicating a strong substrate specificity for the azole class of drugs. There were some differences between these two transporters. The strain expressing *abcC* showed significant resistance to voriconazole (VRC), while the strain expressing *abcF* showed only a slightly increased VRC MIC. The *abcF* strain exhibited significant resistance to econazole (ECON), metconazole (MET), miconazole (MCZ), and prochloraz (PCLZ), while *abcC* did not. Additionally, *abcF* shows a cell wall/membrane sensitivity with significant susceptibility to amphotericin B (AMB), Congo red (CR), and calcofluor white (CW). Strains expressing *abcF*, as well as *abcA, abcC, abcH*, and *abcI*, also showed increased MIC_80_ to rhodamine 6G (R-6G), a known substrate for many ABC transporters (35).

Interestingly, some strains showed more nuanced substrate recognition possibly due to characteristics of the drug such as size, charge, or structure. For example, *abcI* provided a strong resistance phenotype against FLC but shows no resistance to other azoles. Similarly, *abcA* provides resistance to POS but no other azoles.

*abcG* provided no significant (4-fold) change in the MIC_80_ to any of the drugs tested compared to the control strain. This gene may have substrate specificities for compounds not yet tested, or its activity may be masked by endogenous S. cerevisiae transporters that are expressed in the background strain.

### Cell wall/membrane susceptibility.

**FIG 2:**
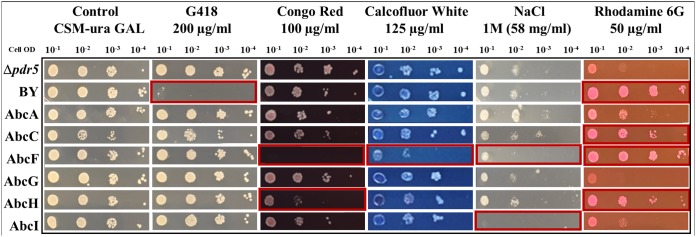
Drug susceptibility agar dot assay. Strains were grown in CSM-ura plus Gal medium to induce plasmid gene expression and then plated in four, 10-fold serial dilutions on CSM-ura plus Gal in the presence of G418, CR, CW, NaCl, and R-6G. Growth on each drug was compared to that on the control CSM-ura plus Gal plate. Red boxes outline strains that differ from the BΔ*PDR5* background strain for that condition.

Stress sensitivity assays on solid medium were performed (Fig. 2) for the strains expressing A. fumigatus transporters with the cell wall or cell membrane stressors Congo red (CR), calcofluor white (CW), and NaCl, as well as for the ABC transporter substrate rhodamine 6G (R-6G) and the selectable antibiotic G418. These serial dilution and growth assays are useful for testing susceptibilities to compounds that either do not solubilize well at high concentrations (NaCl) or to colored compounds that may interfere with broth microdilution spectroscopic readings (CR, CW, and R-6G). G418 resistance due to the kanamycin resistance (Kan^r^) deletion cassette is confirmed in the *PDR5* deletion strains, whereas the wild-type BY4741 strain does not contain the Kan^r^ cassette. The dot assay confirms the susceptibility of the strain expressing *abcF* to CR and CW and is consistent with the MIC_80_ results for those compounds. The dot assay also revealed CR susceptibility for the strain expressing *abcH* that was not seen with the broth microdilution assay. There was a growth deficiency for all strains at the NaCl concentration tested but a particular hypersusceptibility to NaCl for strains expressing *abcF* and *abcI*. Finally, strains expressing *abcC*, *abcF*, and *abcH*, compared to the *ΔPDR5* background strain, showed an increased resistance to the fluorescent dye R-6G. This suggests that these proteins transport R-6G similar to *PDR5*.

### Drug efflux.

**FIG 3:**
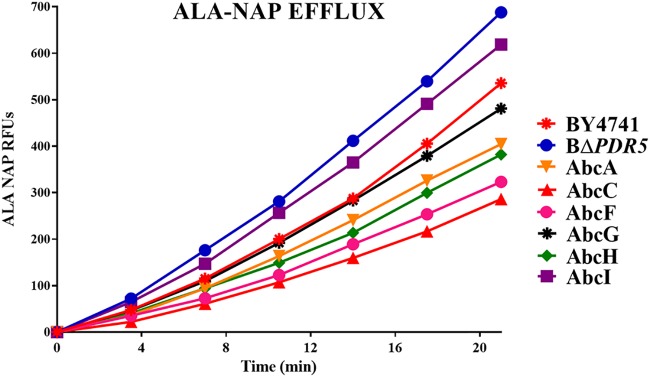
Ala-Nap efflux. Plasmid gene expression was induced by overnight growth in +Gal medium. Fluorescence was measured immediately upon treatment with 128 μg/ml Ala-Nap in glucose-replete buffer. The increase in fluorescence produced by intracellular Ala-Nap hydrolysis over 20 min was measured for each strain in RFU. High levels of fluorescence indicate poor Ala-Nap efflux activity.

**FIG 4:**
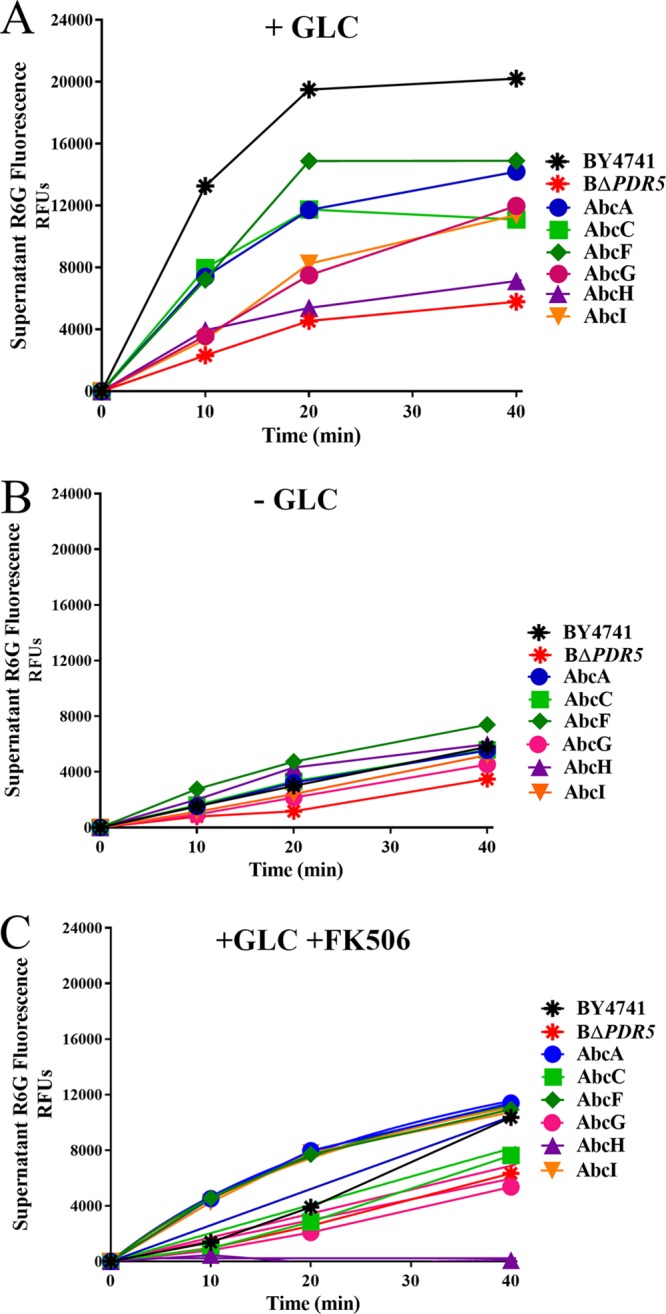
Rhodamine 6G efflux and inhibition by FK506. Plasmid gene expression was induced by overnight growth in galactose medium. Cells were preloaded with R-6G, and the efflux assay was then performed under either glucose-replete (+GLC) conditions (A) or glucose-starved (−GLC) conditions (B) or under glucose-replete conditions with 5 μM FK506 (+GLC +FK506) (C). Fluorescence accumulation in the medium over time was measured in RFU. High levels of fluorescence indicate strong R-6G efflux activity.

**FIG 5:**
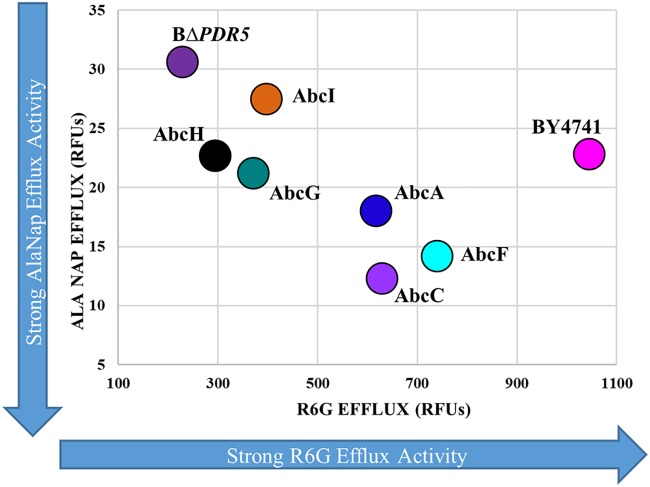
Efflux activity distribution map. Slopes of Ala-Nap and R-6G efflux from 0 to 20 min were plotted for each strain. Low Ala-Nap fluorescence indicates strong efflux activity. High R-6G fluorescence indicates strong efflux activity. Fluorescence is recorded in RFU.

Fluorescence-based assays of pump activity are a useful way to measure and compare the activities of ABC transporters by measuring the fluorescent signal as the dye is transported into the supernatant. We used alanine β-naphthylamide (Ala-Nap) and R-6G fluorescent efflux assays to compare efflux activities between the wild-type strain (BY4741) and the BΔ*PDR5* mutant carrying either an empty plasmid or plasmids with each of the A. fumigatus genes (*abcA, abcC, abcF, abcG, abcH*, or *abcI*) (Fig. 3 and 4) and calculated the rate of efflux for each dye (Tables 2 and 3 and Fig. 5).

**TABLE 2:** Ala-Nap and R-6G energy-dependent efflux rates up to 20 minutes^*a*^

Strain by efflux assay type^*b*^	Efflux rate (RFU/min) from:	
	0–20 min	0–20 min plus FK506
Ala-Nap		
AbcC	12	
AbcF	14	
AbcA	18	
AbcG	21	
BY4741 (WT)	23	
AbcH	23	
AbcI	28	
BΔ*PDR5*	31	
R-6G		
BY4741 (WT)	1,045	184
AbcF	740	366
AbcC	629	247
AbcA	617	409
AbcI	397	398
AbcG	371	99
AbcH	295	4
BΔ*PDR5*	229	122

a Strains are listed in order of strongest to weakest efflux activity for each fluorescent substrate.

b WT, wild type.

**TABLE 3:** ABC transporter activity summary

Gene name	Resistance^*a*^	Hypersusceptibility^*a*^	% change for:		
			Ala-Nap efflux^*b*^^,^^*d*^	R-6G efflux^*b*^^,^^*d*^	R-6G efflux with FK506^*c*^^,^^*d*^
*abcA*	POS, R-6G	—	+72	+169	−34
*abcC*	CLT, FLC, ITC, KTC, POS, R-6G, TEBZ, VRC	—	+158	+175	−61
*abcF*	CLT, ECON, FLC, ITC, KTC, MET, MCZ, PCLZ, POS, R-6G, TEBZ	AMB, CR, CW, NaCl	+121	+223	−51
*abcG*	—	—	+48	+62	−73
*abcH*	R-6G	CR	+35	+29	−99
*abcI*	FLC, R-6G	NaCl	+11	+73	0
WT/*PDR5*	CLT, FLC, ITC, KTC, MET, PCLZ, POS, R-6G, VRC	G418	+35	+356	−82

a As determined by MIC or spot assays to differ from the S. cerevisiae Δ*PDR5* mutant by >4-fold. —, MIC or spot assay value does not significantly differ from the *S. cerevisiae* Δ*PDR5* mutant value.

b Percent increase of efflux compared to the S. cerevisiae Δ*PDR5* mutant.

c Percent decrease in R-6G efflux in the presence of FK506 and GLC compared to GLC-only.

d Based on efflux rates from 0 to 20 min.

Ala-Nap is a fluorescent substrate of ABC transporters as well as transporters of the major facilitator superfamily, which use the proton gradient of the membrane to efflux drugs (36). As Ala-Nap enters the cells, it is cleaved to form the fluorescent molecule β-naphthylamine (37). The more Ala-Nap that is allowed to enter the cells, the more fluorescence is produced. Thus, cells with strong efflux activity yield less fluorescence over time, while cells with poor efflux activity yield higher fluorescence over time.

Figures 3 and 5 and Tables 2 and 3 show the results of the Ala-Nap efflux assay for each strain. Strains expressing *abcC* and *abcF* showed the strongest Ala-Nap efflux, illustrated by the low levels of fluorescence produced over the course of the assay. Strains expressing *abcA*, *abcG*, and *abcH* showed Ala-Nap efflux activity similar to that of the wild-type strain expressing the endogenous *PDR5*. The strain expressing *abcI* as well as the *PDR5* deletion strain produced the highest levels of fluorescence, indicating poor efflux of the substrate.

R-6G is a fluorescent substrate of energy-dependent efflux transporters, mostly ABC transporters. High supernatant fluorescence correlates to strong ABC transporter efflux activity. Figure 4 and Tables 2 and 3 show the results of the R-6G efflux assay comparing efflux in the strains.

When ABC transporters were provided ATP from glucose (+GLC), the wild-type strain (BY4741) as well as the strains expressing each of the A. fumigatus transporter genes (*abcA, abcC, abcF, abcG, abcH*, or *abcI*) showed rapid and measurable R-6G efflux into the medium (Fig. 4A). However, in an energy-depleted state, ABC transporters are inactive, and there is very little R-6G efflux from the cells into the medium (Fig. 4B). The wild-type strain with endogenous Pdr5 had the highest R-6G efflux activity. The *PDR5* deletion strain had the least efflux activity. The *PDR5* deletion strains expressing the A. fumigatus genes showed different levels of energy-dependent R-6G efflux activity, with *abcF* showing the most R-6G efflux activity and *abcH* showing the least activity (Fig. 4 and Table 2; summarized in Table 3).

### Effect of the PDR5-inhibitor FK506.

Cotreatment of an infection with both a primary antifungal agent, such as an azole, and a supplemental agent, such as an efflux inhibitor, has been proposed as a method of combination therapy to combat drug-resistant infections (31, 38–41). FK506 has been shown to be a possible inhibitor of *PDR5*-like ABC transporters (42, 43).

We further analyzed FK506 for its ability to inhibit the active efflux of R-6G in strains overexpressing ABC transporters by treating the cells with FK506 during the R-6G efflux assay (Fig. 4C and Table 3). The wild-type strain (BY4741) and strains expressing the six A. fumigatus genes were treated with 5 μM FK506 to determine if this drug showed a measurable inhibitory effect on R-6G efflux. Many of the FK506-treated samples had dramatically reduced R-6G efflux compared to that with the untreated samples (+GLC versus +GLC +FK506), as shown in Fig. 4A and C and summarized in Table 3. R-6G efflux in strains expressing *abcC, abcF, abcG*, or *abcH* was reduced by greater than 50% in the presence of FK506. The strain expressing *abcA* was less inhibited than were other strains by the addition of FK506 to the R-6G efflux assay, while the strain expressing *abcI* was completely uninhibited by FK506 (Fig. 4C and Tables 2 and 3). This is possibly due to some variation at the transporters’ FK506 binding site or R-6G binding site that determines the degree of transporter inhibition in these particular transporters. Alternatively, *abcA* and *abcI* may not share a certain feature of the canonical Pdr5-like transporter that FK506 usually recognizes.

### Efflux pump gene expression in A. fumigatus clinical isolates.

**FIG 6:**
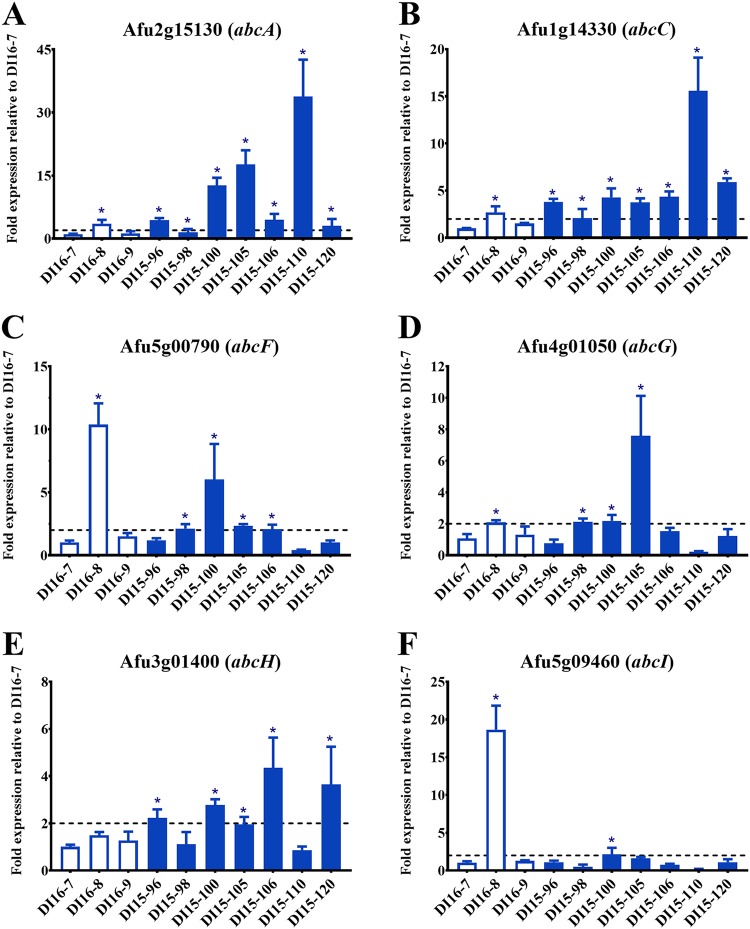
Relative expression of efflux pump-encoding genes among A. fumigatus clinical isolates by RT-qPCR. Expression of each gene of interest relative to the expression observed in the triazole-susceptible clinical isolate DI16-7. Bars denoting the relative expression observed for triazole-susceptible isolates are shown in white, and bars denoting the relative expression observed for triazole-resistant isolates are shown in blue. The dotted line delineates 2-fold greater expression than that in the control isolate DI16-7. Asterisks highlight mean values that are ≥2-fold greater than that of DI16-7.

The endogenous gene transcript levels of the six putative efflux pumps in A. fumigatus were analyzed in triazole-susceptible and triazole-resistant A. fumigatus isolates (Fig. 6). The clinical isolates were tested for drug susceptibility to various azoles (VRC, ISV, ITC, and POS) and determined to be susceptible based on VRC MICs of 0.25 to 0.5 μg/ml (see Table S3 in the supplemental material). The expression levels of *abcA, abcC, abcF, abcG, abcH*, or *abcI* in all clinical isolates were compared to those in the triazole-susceptible isolate DI16-7 using real-time reverse transcription-quantitative PCR (RT-qPCR) (Fig. 6).

10.1128/mBio.00338-20.5 DATA SET S1 RT-qPCR data set. Download Data Set S1, XLSX file, 0.1 MB. Copyright © 2020 Esquivel et al. 2020 Esquivel et al. This content is distributed under the terms of the Creative Commons Attribution 4.0 International license.

The expression of *abcA* ranged from 1.2- to 33.8-fold that of the comparator isolate DI16-7, with 5 of the 6 resistant isolates exhibiting 2-fold greater *abcA* expression than that of DI16-7.

The expression of *abcC* ranged from 1.5- to 15.5-fold that of the comparator isolate DI16-7, with all of the resistant isolates exhibiting 2-fold greater *abcC* expression than that of DI16-7.

The expression of *abcF* ranged from 0.4- to 10.4-fold that of the comparator isolate DI16-7. While 4 of the 6 resistant isolates exhibited 2-fold greater *abcF* expression than that of DI16-7, the highest *abcF* expression was in the susceptible isolate DI16-8, at 10.4-fold that of DI16-7.

The expression of *abcG* ranged from 0.2- to 7.6-fold that of the comparator isolate DI16-7, with 3 of the 6 resistant isolates exhibiting 2-fold greater *abcG* expression than that of DI16-7.

The expression of *abcH* ranged from 0.9- to 4.4-fold that of the comparator isolate DI16-7, with 4 of the 6 resistant isolates exhibiting 2-fold greater *abcH* expression than that of DI16-7.

The expression of *abcI* ranged from 0.2- to 18.7-fold that of the comparator isolate DI16-7. Only 1 of the 6 resistant isolates exhibited 2-fold greater *abcI* expression than that of DI16-7, while the susceptible isolate DI16-8 had the highest expression level, at 18.7-fold that of DI16-7.

## DISCUSSION

The efflux of antifungal drugs via ABC transporters constitutes a major cause of clinical multidrug resistance. The objectives of this study were to identify A. fumigatus plasma membrane ABC transporters that may contribute to antifungal drug resistance and to characterize the substrates and efflux properties of the individual pumps when expressed in the model organism S. cerevisiae. The A. fumigatus genome contains many putative ABC transporters, and determining which of these genes are transcribed and translated into functional efflux pumps will improve gene annotation databases and help researchers gain more insight into fungal evolutionary relationships.

In addition, the development of efflux inhibitors is a potential strategy to overcome antifungal resistance, especially when given as cotherapy with fungus-specific drug treatments (12, 31, 38, 43–45). Therefore, increased emphasis on inhibitors of efflux should be considered in the development of new therapeutic strategies for aspergillosis.

We previously showed evidence for intracellular accumulation of radioactively labeled FLC in A. fumigatus, as well as energy-dependent azole efflux (29). This illustrated that ABC transporters in A. fumigatus may play a role in azole drug resistance and that transport or intracellular accumulation of FLC is something that could be manipulated with antifungal treatments. Identifying A. fumigatus transporters that have substrate specificity for common antifungals would help foster the development of compounds to be used as efflux inhibitors.

Clorgyline and FK506 are just two examples of potential broad-spectrum compounds that may reverse azole resistance in fungi. Supporting this role of clorgyline and FK506, we found that the addition of clorgyline to A. fumigatus samples treated with FLC caused an increased accumulation of FLC inside the cells (Fig. 1 and Table 3). We also found that the addition of FK506, even at sub-MICs, has an inhibitory effect on the active efflux of R-6G in the S. cerevisiae strains expressing A. fumigatus transporters (Fig. 4 and Tables 3 and 4).

**TABLE 4:** A. fumigatus putative ABC transporter genes analyzed in this study and their orthologs in other fungi^*a*^

Gene name	Ortholog gene ID for:						
	Aspergillus fumigatus	Aspergillus nidulans	Aspergillus niger	Neurospora crassa	Candida albicans	Saccharomyces cerevisiae	Saccharomyces pombe
*abcA*	Afu2g15130	AN10949	An15g02930	NCU05591	*CDR1*	*PDR15*	*bfr1*
*abcC*	Afu1g14330	AN0771	An01g12380	NCU05591	*CDR4*	*PDR15*	*bfr1*
*abcF*	Afu5g00790	AN8344	An05g01660	NCU05991	*CDR4*	*PDR5*	*bfr1*
*abcG*	Afu4g01050	AN8489/*atrB*	An14g02610	mig-12	*SNQ2*	*SNQ2*	*bfr1*
*abcH*	Afu3g01400	AN8489/*atrB*	An13g0360	mig-12	*SNQ2*	*SNQ2*	*bfr1*
*abcI*	Afu5g09460	AN8813	An11g02110	NCU10009	*SNQ2*	*SNQ2*	*bfr1*

a Orthologs were determined using the *Aspergillus* Genome Database (AspGD; http://www.aspgd.org/).

Our characterization of the A. fumigatus putative ABC transporters AbcA, AbcC, AbcF, AbcG, AbcH, and AbcI expressed in S. cerevisiae has shown interesting differences in substrate specificity and energy-dependent efflux. Strains expressing the genes demonstrated various degrees of resistance to an assortment of drugs that are thought to be efflux pump substrates (Table 1; summarized in Table 3).

AbcC and AbcF provided resistance to many azoles and showed substrate specificity similar to that of endogenous Pdr5. AbcA and AbcI had a very narrow substrate specificity that included POS and FLC, respectively. The drug susceptibility results suggest that the *abcA, abcC, abcF, abcH*, and *abcI* genes encode functional energy-dependent ABC transporters that can in some cases compensate for the S. cerevisiae Pdr5 transporter to increase resistance to various drugs. These findings confirm a potential role for these A. fumigatus transporters in drug resistance and can begin to identify each transporter’s substrate specificity.

While we focused our substrate specificity analysis on commonly used antifungal compounds, these transporters may have an entirely different set of efflux substrates. such as sterols, lipids, detergents, peptides, sugars, and amino acids. For example, AbcG had more subtle effects on drug resistance in the strain for the compounds tested but exhibited efflux activity of both R-6G and Ala-Nap, indicating that AbcG is expressed and functional. Continued exploration of drug-resistant phenotypes of our strains along with bioinformatics comparisons of the transporters’ sequences and predicted structures may illuminate important evolutionary events and even reveal potential efflux inhibitor drug targets.

The Ala-Nap and R-6G efflux assays provided even more distinguishing phenotypes between the A. fumigatus transporters (Fig. 3 and 5 and Tables 2 and 3). There were transporters that had strong R-6G efflux activity (AbcA, AbcC, and AbcF) and others that showed less R-6G efflux (AbcG, AbcH, and AbcI). It is noteworthy that the strain expressing *abcI* exhibited the least efflux of Ala-Nap, whereas the strain expressing *abcH* exhibited the least efflux as measured by R-6G, again emphasizing differing substrate specificities. Additionally, all of the A. fumigatus transporters except AbcI showed an impairment in R-6G efflux activity in response to treatment with the efflux inhibitor FK506 (Fig. 4C and Tables 2 and 3).

We then demonstrated that each of the 6 genes of interest is differentially expressed in a collection of triazole-susceptible and -resistant A. fumigatus isolates (Fig. 6). We observed that the expression levels of the ABC-type efflux pump-encoding genes *abcA, abcC*, and *abcH* were higher among the triazole-resistant isolates than among the susceptible isolates. In contrast, *abcI* was only overexpressed in a single isolate, which was triazole susceptible. AbcI had a narrow substrate specificity that included fluconazole and R-6G, and as stated previously, the transporters may have pump functions that do not involve azoles. *abcF* and *abcG* were overexpressed in both triazole-resistant and -susceptible isolates.

Others have shown A. fumigatus to have phenotypic plasticity, a characteristic that allows it to thrive in a variety of environments by making rapid adjustments to the transcriptome after drug exposure (46). It might be important to measure the expression levels of the six transporter genes in these isolates after exposure to antifungals, as transporter transcription may be induced under these conditions (28).

In addition to phenotypic plasticity, there seems to be much redundancy in the A. fumigatus genome, especially with regard to transporter genes (47), so that the effect of a single transporter may not be enough to change the drug susceptibility profile of an isolate. Instead, the expression of multiple transporters, all with diverse substrate specificities, could very well determine a susceptible isolate from a multidrug-resistant isolate.

Taken together, our work shows that ABC transporters are a clinically important group of transporters that remain understudied and enigmatic in most fungal species. Our results highlight the remarkable diversity, substrate specificity, and evolutionary divergence among the ABC transporters even within the same organism and demonstrate the relevance of efflux pumps as one of the factors of multidrug resistance in A. fumigatus.

Future work should include sequence analyses that distinguish residues important for the transporters’ unique substrate specificity and identify drugs that induce transporter expression, transcription factors that regulate transporter gene expression, and even similarities and differences in the endogenous promoters and terminators of these transporters.

## MATERIALS AND METHODS

### Strains and media.

The strains used in this study and their genotypes are listed in Table S1 in the supplemental material. The media used include YNB (1.7 g/liter yeast nitrogen base without amino acids), CSM (1.7 g/liter yeast nitrogen base without amino acids, 0.8 g/liter complete supplemental mixture), and CSM-ura (1.7 g/liter yeast nitrogen base without amino acids, 0.8 g/liter complete supplemental mixture without uracil).

10.1128/mBio.00338-20.2 TABLE S1 Strains used in this study. Download Table S1, XLSX file, 0.1 MB. Copyright © 2020 Esquivel et al. 2020 Esquivel et al. This content is distributed under the terms of the Creative Commons Attribution 4.0 International license.

### [^3^H]FLC efflux in A. fumigatus.

Mycelial masses (approximately 3 mg dry weight) of A. fumigatus strain AF293 were preloaded with ^3^H-fluconazole (FLC) (19.5 nM) for 24 h, as per the standard radioactive import assay described previously (29). The cells were then washed and resuspended in a 10 ml of YNB medium, and intracellular [^3^H]FLC was evaluated over time under both glucose-energized (with 2% glucose) and deenergized (without glucose) conditions. Radioactivity was quantified by liquid scintillation counting (Beckman Coulter LS 6500) and calculated as the cpm per mg of A. fumigatus biomass as a function of time at the 4-, 8-, and 24-h time points.

To test the effect of clorgyline, we treated energized (2% glucose), efflux-active cells with either [^3^H]FLC alone (19.25 nM) or [^3^H]FLC with 233 μM clorgyline [*N*-methyl-*N*-propargyl-3-(2,4-dichlorophenoxy)propylamine hydrochloride] and compared the results to those with [^3^H]FLC treatment in glucose-starved, efflux inactive cells. Radioactivity was quantified after 24 h of treatment.

Error bars in Fig. 1 represent the standard deviation of the means of biological triplicates for each condition. Differences between sets of samples were evaluated by an unpaired two-tailed Student *t* test. A *P* value of <0.05 was considered significant.

### A. fumigatus RNA isolation, transporter selection, and cDNA synthesis.

RNA was extracted from frozen fungal mycelia using the Qiagen RNeasy minikit for filamentous fungi. The RNA was used for cDNA synthesis using the Thermo Scientific SuperScript II 2 synthesis kit, as directed by the manufacturer's instructions, using a combination of random hexamer and poly(dT) oligonucleotides provided in the kit.

ABC transporters were selected for this analysis by performing an NCBI BLASTp search against A. fumigatus Af293 proteins using the S. cerevisiae Pdr5 protein sequence. The translated sequence and predicted protein domains include two nucleotide binding domains and two transmembrane domains each containing 6 TMD segments. More specifically, genes that are Pdr5-like proteins, with the canonical subfamily G domain orientation (reviewed in references 13 and 17), were selected. Gene-specific oligonucleotides were designed to isolate A. fumigatus genes from the cDNA by PCR with Phusion high-fidelity polymerase (Thermo Scientific). Not all genes were successfully amplified from cDNA, which could indicate that those genes were not expressed under the growth conditions used, but 11 genes were successfully amplified. The oligonucleotides that successfully amplified 11 different A. fumigatus genes from cDNA are listed in Table S2.

10.1128/mBio.00338-20.3 TABLE S2 Oligonucleotides used in this study. Download Table S2, XLSX file, 0.1 MB. Copyright © 2020 Esquivel et al. 2020 Esquivel et al. This content is distributed under the terms of the Creative Commons Attribution 4.0 International license.

### Plasmid construction.

pYES2 is a 5.9-kb vector designed for the expression of recombinant proteins in S. cerevisiae (catalog no. V825-20; Invitrogen). The vector contains the yeast *GAL1* promoter for high-level, inducible protein expression in the presence of galactose (Gal) and a *CYC1* transcriptional terminator.

The 11 isolated A. fumigatus transporter genes were amplified using oligonucleotides designed for homologous recombination into the pYES2 plasmid (Table S2). The plasmid was cut with HindIII and PvuII and dephosphorylated with alkaline phosphatase (New England BioLabs). Homologous recombination of the PCR products and the linearized plasmid was performed in S. cerevisiae wild-type strain W303 using the standard *Saccharomyces* lithium acetate transformation protocol (48).

pYES2 plasmids containing A. fumigatus strain AF293 transporter genes were isolated from ura^+^ colonies. Escherichia coli was transformed with the isolated plasmids using the DH5α heat shock protocol (49) and plated on LB agar containing 100 μg/ml ampicillin. Plasmids were isolated from amp^+^ selected colonies. Plasmids carrying the correct size insert were identified by PCR and by restriction digestion and agarose gel electrophoresis. DNA sequencing confirmed the correct sequence and orientation for six out of the 11 inserted A. fumigatus genes (Afu2g15130 [*abcA*], Afu1g14330 [*abcC*], Afu5g00790 [*abcF*], Afu4g01050 [*abcG*], Afu3g01400 [*abcH*], and Af5109460 [*abcI*]; names in brackets reflect published genes or new names based on the next available letter for *abc*). The genes are listed in Table 4 with orthologues in other well-known fungi. The other five A. fumigatus genes did not match exactly to the gene sequences provided in the *Aspergillus* Genome Database and so were not used further.

### S. cerevisiae background strain construction.

To eliminate the complication of endogenous *PDR5* expression in S. cerevisiae from the analysis of A. fumigatus transporters, the *PDR5* gene was deleted from parental strain BY4741 by homologous recombination, using the pFA6a-kanMX6 deletion cassette (50), resulting in strain BΔ*PDR*5 (described in Table S1 and Fig. S1). The oligonucleotides used for the deletion cassette are listed in Table S2.

10.1128/mBio.00338-20.1 FIG S1 *PDR5* deletion in recipient S. cerevisiae strains causes drug susceptibility. (A and B) Parent S. cerevisiae strains W303 and BY4741 (A) were compared to their *PDR5* deletion derivative strains W*ΔPDR5* and B*ΔPDR5* (B) for fluconazole susceptibility. The deletion of *PDR5* causes drug susceptibility in both strains. W303 and W*ΔPDR* are *ade2* mutants, resulting in their pink color. Download FIG S1, TIF file, 1.7 MB. Copyright © 2020 Esquivel et al. 2020 Esquivel et al. This content is distributed under the terms of the Creative Commons Attribution 4.0 International license.

DNA was isolated from G418-resistant colonies and used for PCR to confirm that the G418 resistance gene had replaced the *PDR5* gene at the *PDR5* locus in the BY4741 *PDR5* deletion strain (BΔ*PDR5*). The oligonucleotides used to confirm the *PDR5* deletion are listed in Table S2.

BΔ*PDR5* was transformed with plasmids containing the A. fumigatus genes for analyses similar to that done by Paul and Moye-Rowley (51). We expect all proteins to be expressed at similar levels because the cloned genes were all under the control of the same *GAL1* promoter, the *CYC1* terminator, from the same pYES2 plasmid. They were induced at the same temperature, for the same amount of time, and with identical galactose concentrations. Most importantly, the pump genes were expressed in the same S. cerevisiae background strain which would induce *GAL1* expression at the same level in response to galactose. Independently isolated individual transformants from the same transformation had the same phenotype, confirming that there was no copy number effect.

All experiments were performed multiple times on different days from different single colonies. The results for a single strain between multiple experiments are consistent and repeatable, indicating that the plasmid copy number is not extremely varied between experiments or between strains. The cells had doubling times in galactose nearly identical to those of the parent S. cerevisiae strain BY4741 and even slightly faster doubling time in galactose than with the *PDR5*-deleted background strain BΔ*PDR*5 (data not shown). Hence, the expression of the cloned genes does not negatively affect cell growth or viability.

### MIC drug susceptibility tests.

The MICs of compounds for recombinant S. cerevisiae strains were determined in accordance with the CLSI M27-A3 broth microdilution method (52), except the method was modified by using a CSM-ura medium to retain the plasmid. Briefly, S. cerevisiae strains were inoculated in microtiter plates in the presence of a series of 2-fold dilutions of a variety of compounds and antifungal drugs. The plates were incubated in CSM-ura plus 2% Gal (plasmid expression induced) at 30°C for 72 h to account for the slower growth in galactose. The MIC_80_ is the drug concentration at which growth of cells was inhibited by at least 80% compared to the growth found for a no-drug control as determined by the optical density at 600 nm (OD_600_) with a microplate reader (BioTek).

The drugs used for MIC testing include amphotericin B (AMB), cerulenin (CER), caspofungin (CFG), cycloheximide (CH), chloramphenicol (CHL), clotrimazole (CLT), Congo red (CR), calcofluor white (CW), ebselen (EBS), econazole (ECON), fenpropimorph (FEN), fluconazole (FLC), G418, isavuconazole (ISV), itraconazole (ITC), ketoconazole (KTC), metconazole (MET), miconazole (MCZ), 4-nitroquinolone (4-NQ), nystatin (NYT), prochloraz (PCLZ), posaconazole (POS), rhodamine 6G (R-6G), tebuconazole (TEBZ), terbinafine (TERB), tetracycline (TETCL), and voriconazole (VRC).

### Stress sensitivity serial dilution spot assay.

Strains were grown overnight in CSM-ura medium with 2% Gal. The strains were dotted on agar plates starting at an OD_600_ of 0.1 and then diluted in three 10-fold serial dilutions of decreasing cell concentrations from left to right for each condition tested (Fig. 2). The drugs tested were G418 (200 μg/ml), CR (100 μg/ml), CW (125 μg/ml), NaCl (1 M), and R-6G (50 μg/ml). The drugs were added to CSM-ura plus Gal agar after the medium had been autoclaved and before pouring the plates. A plate with no added drug was used as a growth control for comparison. All plates were incubated for 72 h at 30°C and then imaged. A reduction in colony numbers and/or growth compared to the control were considered an increase in sensitivity.

### Ala-Nap efflux assay.

An alanine β-naphthylamide (Ala-Nap) fluorescence assay was performed as previously described (53). Strains were grown to exponential phase in CSM-ura plus 2% Gal. Cells were washed and diluted to an OD_600_ of 0.6 in phosphate-buffered saline (PBS) with or without 4% glucose, and 100 μl of the cultures was added to the wells of black flat-bottom microtiter plates (Costar 3603).

The reaction was initiated by the addition of 100 μl Ala-Nap (Sigma-Aldrich) to a final concentration of 128 μg/ml. Fluorescence was quantified at intervals of 3 min 30 s at an excitation wavelength of 320 nm and an emission wavelength of 460 nm over 1 h at 35°C using a fluorescent plate reader (BioTek). The data are presented in relative fluorescence units (RFU). All experiments were performed in biological triplicates, and the average value was plotted for each time point in Fig. 3. The slopes of the efflux in the individual strains in the presence of glucose were calculated for all strains plotted on a graph up to 20 min in Fig. 5.

### Rhodamine 6G efflux assay.

A. fumigatus ABC efflux transporters were analyzed for potential functionality using a microtiter assay of the fluorescent dye rhodamine 6G (R-6G), which measures R-6G efflux from cells into the supernatant over time. Strains were initially grown in 50 ml CSM-ura plus 2% Gal to exponential phase, and the R-6G assay was performed as previously described (53). Briefly, cells were preloaded with R-6G at a final concentration of 10 μM (4.8 μg/ml), which is well below the R-6G MIC for S. cerevisiae strain BΔ*PDR5.* The cells were washed and then resuspended in PBS with or without glucose. Efflux of the R-6G into the supernatant was measured at time points 0 (immediately after the dye-loaded cells were resuspended in PBS), as well as after 10, 20, and 40 min.

For R-6G efflux assays with FK506, the samples were preloaded with R-6G as described above and allowed to incubate for 30 min. FK506 was then added at a final concentration of 5 μM (4 μg/ml) to the sample, which is well below the FK506 MIC for S. cerevisiae strain BΔ*PDR5.* The cells were allowed to coincubate with the R-6G for another 30 min. The samples were washed and resuspended in fresh PBS with glucose and 5 μM (4 μg/ml) FK506, and supernatant samples were taken immediately and at the time points indicated in Fig. 4.

The data are presented as relative fluorescence units (RFU). All experiments were performed in biological triplicates, and the average value was plotted for each time point in Fig. 4. The slopes of the efflux in the individual strains in the presence of glucose were calculated for all strains plotted on a graph up to 20 min in Fig. 5.

### RT-qPCR assessment of gene expression in A. fumigatus clinical isolates.

Conidia from 10 clinical isolates of A. fumigatus, composed of 7 previously characterized triazole-resistant isolates and 3 triazole-susceptible isolates (described in Table S3), were incubated at 37°C on an orbital shaker at 250 rpm in *Aspergillus* minimal medium overnight (54, 55). As previously described, RNA was then extracted from mature hyphae following a liquid nitrogen crush, and cDNA was synthesized from 500 ng total RNA using the RevertAid reverse transcriptase (RT) kit (Thermo Scientific) (56). The A. fumigatus genes Afu1g10910 (*tubA*), Afu2g15130 (*abcA*), Afu1g14330 (*abcC*), Afu5g00790 (*abcF*), Afu4g01050 (*abcG*), Afu3g01400 (*abcH*), and Afu5g09460 (*abcI*) were amplified from cDNA using the primers listed in Table S2, PCR master mix, and SYBR green, according to the manufacturer’s instructions (Applied Biosystems). The PCR conditions were 95°C for 10 min for AmpliTaq Gold activation, 95°C for 15 s for denaturation, and 60°C for annealing/extension for 40 cycles. The dissociation curve and threshold cycle (*C_T_*) were determined using the 7500 detection real-time PCR system (Applied Biosystems). The 2^−ΔΔ^*^CT^* method was then used to calculate changes in gene expression, using *tubA* as the housekeeping gene and the triazole-susceptible clinical isolate DI16-7 as the comparator to determine the relative expression of each efflux pump-encoding gene of interest. Reactions were performed in triplicate from biological triplicates. As previously described, Δ*C_T_* values were used to calculate the standard error (57).

10.1128/mBio.00338-20.4 TABLE S3 A. fumigatus triazole-susceptible and -resistant clinical isolates. Download Table S3, XLSX file, 0.1 MB. Copyright © 2020 Esquivel et al. 2020 Esquivel et al. This content is distributed under the terms of the Creative Commons Attribution 4.0 International license.
