# Supplementary figures and images for: Characterization of the Efflux Capability and Substrate Specificity of Aspergillus fumigatus PDR5-like ABC Transporters Expressed in Saccharomyces cerevisiae
Source: mBio. 2020 Mar 24;11(2):e00338-20. doi: 10.1128/mBio.00338-20 (PMC7157516; doi:10.1128/mBio.00338-20)

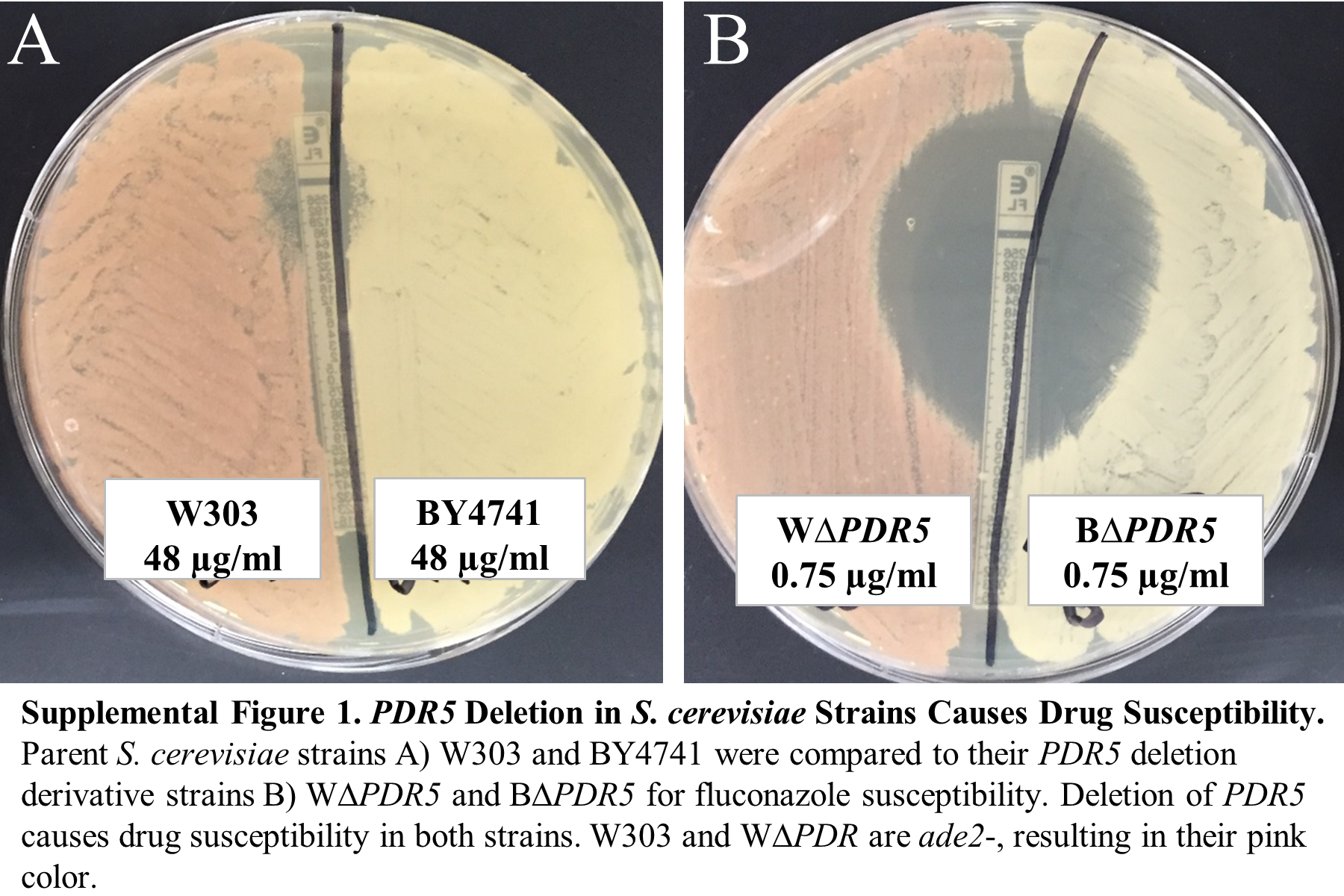

Supplement: FIG S1 [file mBio.00338-20-sf001.tif]
